# Supplementary material for: In Silico tool for predicting, designing and scanning IL-2 inducing peptides
Source: Sci Rep. 2025 Jul 16;15:25692. doi: 10.1038/s41598-025-08388-2 (PMC12267595; doi:10.1038/s41598-025-08388-2)
Supplement: Supplementary file 2 — Supplementary Material 2. [file 41598_2025_8388_MOESM2_ESM.docx]

**Supplementary Information**

**Figure SF1:** Histogram plot showing the frequency distribution of peptide length of the alternate dataset 1.

**
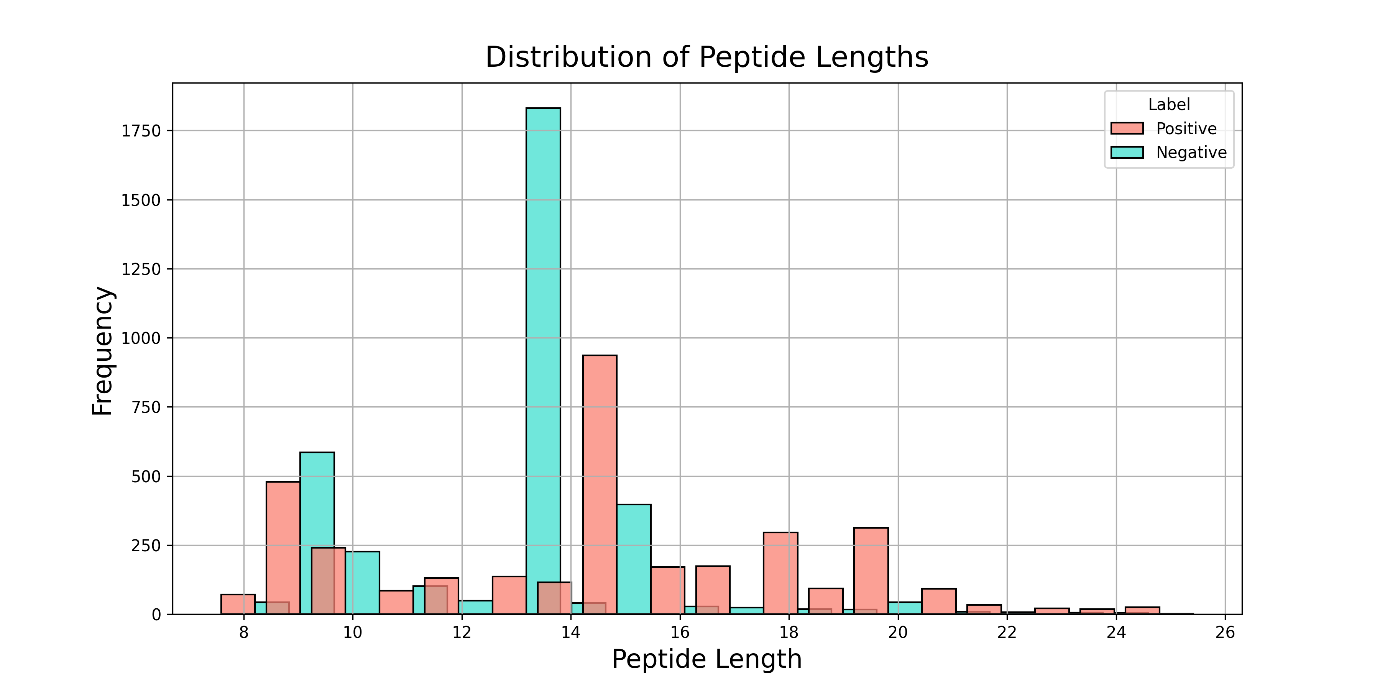
**

**Figure SF2:** Histogram plot showing the frequency distribution of peptide length of the alternate dataset 2.

**
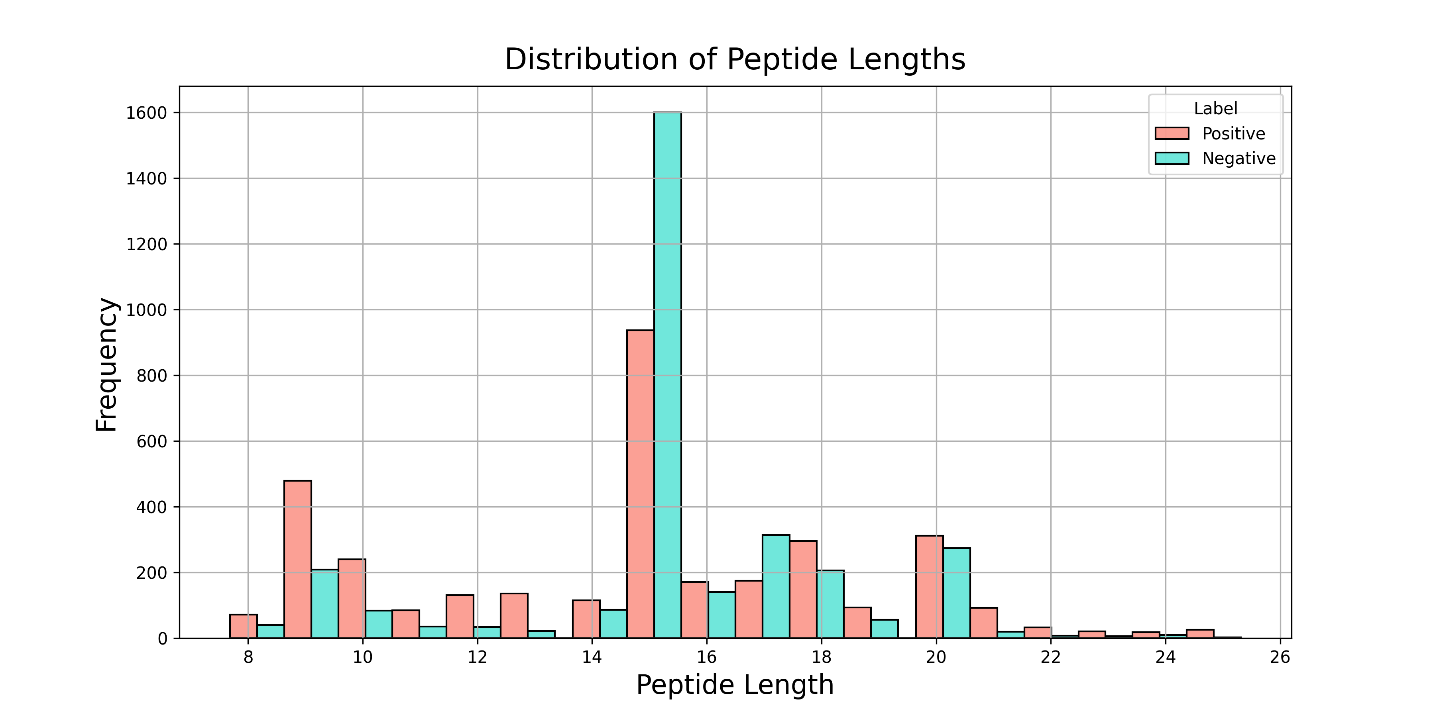
**

**Figure SF3:** Bar plot of the peptides' average single amino acid composition in the IL-2 inducers and non-inducers of the alternate dataset 1. The adjusted p-values are shown above the bars.


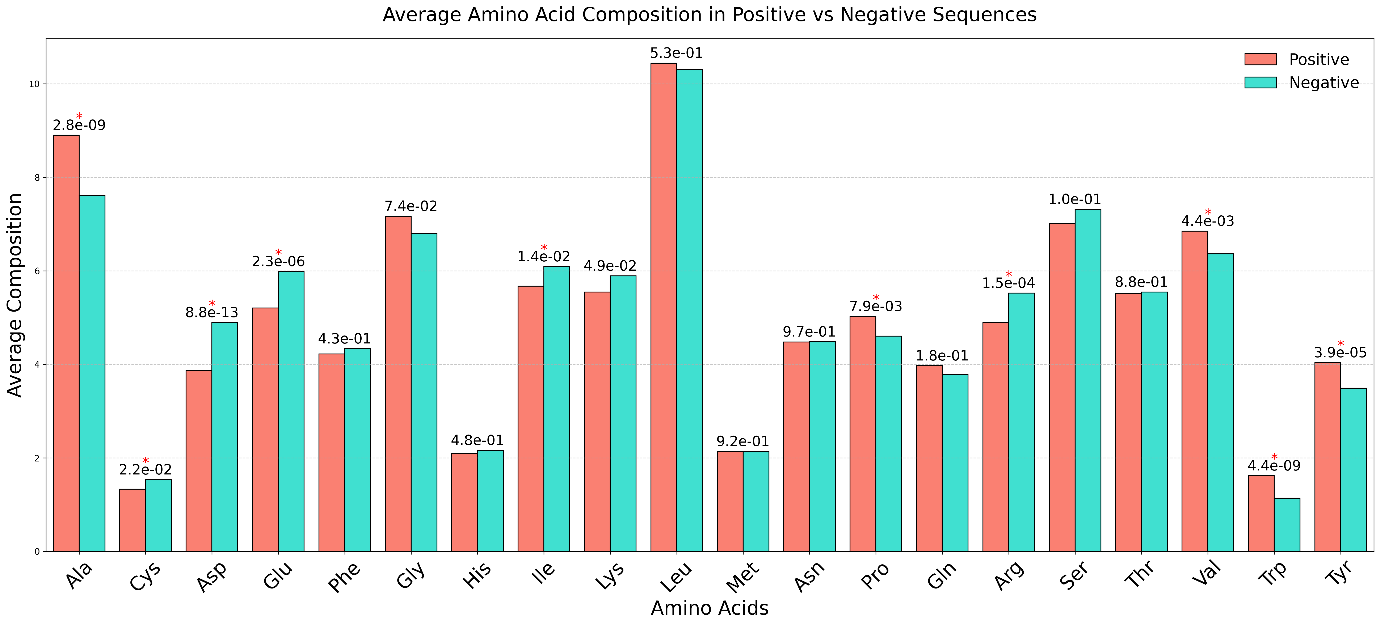


**Figure SF4:** Bar plot of the peptides' average single amino acid composition in the IL-2 inducers and non-inducers of the alternate dataset 2. The adjusted p-values are shown above the bars.


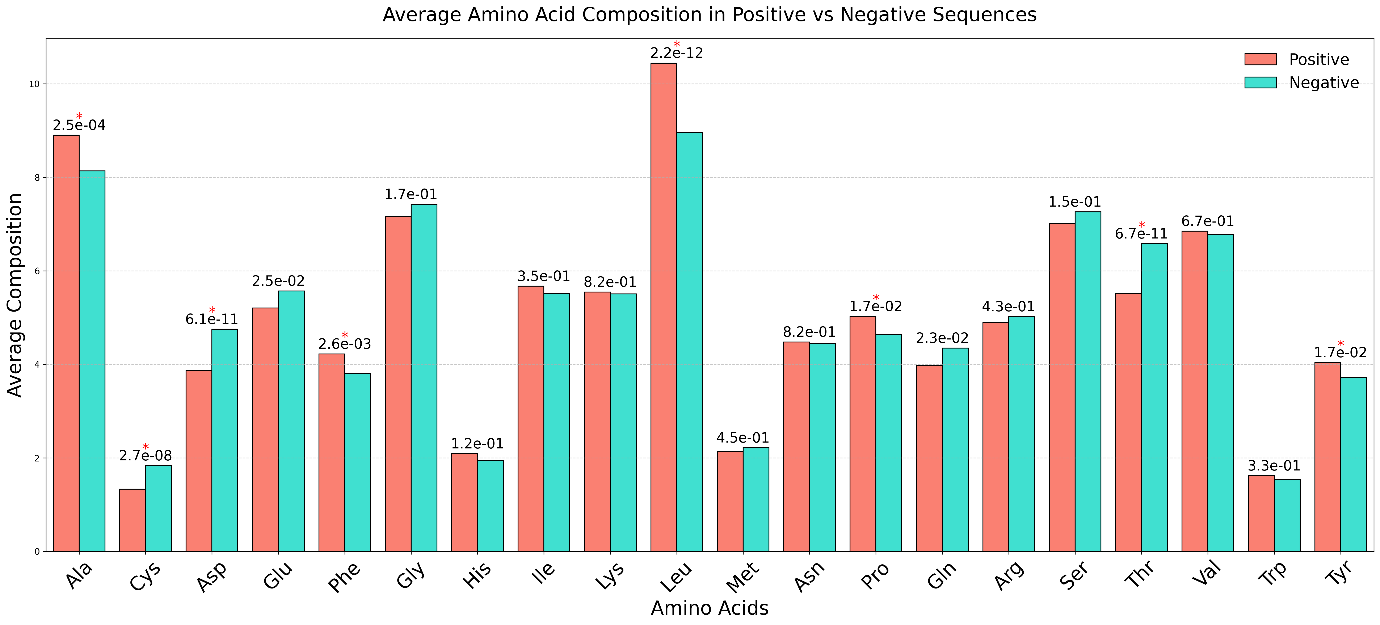


**Figure SF5:** Two-sample logo displaying the positional conservation of amino acid for the alternate dataset 1.

**
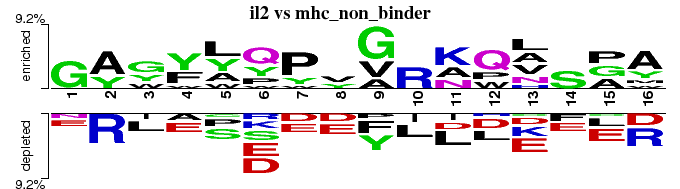
**

**Figure SF6:** Two-sample logo displaying the positional conservation of amino acid for the alternate dataset 2.

**
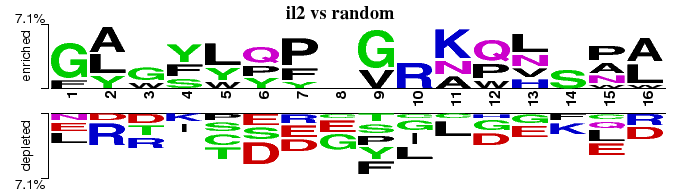
**
